# Cefepime-induced neurotoxicity: a systematic review

**DOI:** 10.1186/s13054-017-1856-1

**Published:** 2017-11-14

**Authors:** Lauren E. Payne, David J. Gagnon, Richard R. Riker, David B. Seder, Elizabeth K. Glisic, Jane G. Morris, Gilles L. Fraser

**Affiliations:** 1 University of New England College of Pharmacy, 716 Stevens Ave, Portland, ME 04102 USA; 2 Department of Pharmacy, Maine Medical Center, 22 Bramhall St, Portland, ME 04102 USA; 3 Department of Critical Care, Maine Medical Center, 22 Bramhall St., Portland, ME 04102 USA; 4 Maine Medical Partners Neurology, 49 Spring St, Scarborough, ME 04074 USA; 5 Department of Pharmacy and Critical Care, Maine Medical Center, 22 Bramhall St, Portland, ME 04102 USA

**Keywords:** Adverse events, Blood–brain barrier, Cefepime, Cephalosporin, Coma, Intensive care units, Myoclonus, Seizures, Status epilepticus

## Abstract

**Background:**

Cefepime is a widely used antibiotic with neurotoxicity attributed to its ability to cross the blood–brain barrier and exhibit concentration-dependent ϒ-aminobutyric acid (GABA) antagonism. Neurotoxic symptoms include depressed consciousness, encephalopathy, aphasia, myoclonus, seizures, and coma. Data suggest that up to 15% of ICU patients treated with cefepime may experience these adverse effects. Risk factors include renal dysfunction, excessive dosing, preexisting brain injury, and elevated serum cefepime concentrations. We aimed to characterize the clinical course of cefepime neurotoxicity and response to interventions.

**Methods:**

A librarian-assisted search identified publications describing cefepime-associated neurotoxicity from January 1980 to February 2016 using the CINAHL and MEDLINE databases. Search terms included cefepime, neurotoxicity, encephalopathy, seizures, delirium, coma, non-convulsive status epilepticus, myoclonus, confusion, aphasia, agitation, and death. Two reviewers independently assessed identified articles for eligibility and used the Preferred Reporting Items for Systematic review and Meta-Analysis Protocols (PRISMA-P) for data reporting.

**Results:**

Of the 123 citations identified, 37 (representing 135 patient cases) were included. Patients had a median age of 69 years, commonly had renal dysfunction (80%) and required intensive care (81% of patients with a reported location). All patients exhibited altered mental status, with reduced consciousness (47%), myoclonus (42%), and confusion (42%) being the most common symptoms. All 98 patients (73% of cohort) with electroencephalography had abnormalities, including non-convulsive status epilepticus (25%), myoclonic status epilepticus (7%), triphasic waves (40%), and focal sharp waves (39%). As per Food and Drug Administration (FDA)-approved dosing guidance, 48% of patients were overdosed; however, 26% experienced neurotoxicity despite appropriate dosing. Median cefepime serum and cerebrospinal fluid (CSF) concentrations were 45 mg/L (n = 21) and 13 mg/L (n = 4), respectively. Symptom improvement occurred in 89% of patients, and 87% survived to hospital discharge. The median delay from starting the drug to symptom onset was 4 days, and resolution occurred a median of 2 days after the intervention, which included cefepime discontinuation, antiepileptic administration, or hemodialysis.

**Conclusions:**

Cefepime-induced neurotoxicity is challenging to recognize in the critically ill due to widely varying symptoms that are common in ICU patients. This adverse reaction can occur despite appropriate dosing, usually resolves with drug interruption, but may require additional interventions such as antiepileptic drug administration or dialysis.

## Background

The neurotoxic effects of cefepime, a fourth-generation cephalosporin antibiotic, were first reported in 1999 [1]. The mechanism for these adverse events is not fully understood, but is thought to be related to concentration-dependent competitive ϒ-aminobutyric acid (GABA) antagonism [2]. Symptoms are often associated with decreased cefepime clearance in the setting of reduced glomerular filtration, and increased central nervous system penetration secondary to blood–brain barrier (BBB) dysfunction [2, 3]. Data suggest that the primary risk factor for cefepime neurotoxicity is renal dysfunction, particularly when dosing is not appropriately reduced [4, 5].

Fugate et al. suggested that up to 15% of ICU patients will experience one or more neurotoxic symptoms, but because these symptoms are common in the critically ill, recognition that these adverse events are drug-related may be delayed, predisposing patients to further toxicity [5]. The high frequency of renal impairment affecting ICU patients and the difficulties in quantifying renal dysfunction when creatinine-based equations are used may also contribute [4, 6, 7] since Food and Drug Administration (FDA) dosing recommendations rely on estimates of creatinine clearance (CrCl) [3]. In addition, ICU patients are prone to disruptions in BBB integrity associated with systemic inflammation, resulting in greater penetration of cefepime into the central nervous system (CNS). Approximately 10% of serum cefepime crosses the BBB; however, renal impairment, decreased protein binding, and increased organic acid accumulation can increase this transfer up to 45% [2, 8].

Missing from the literature is a comprehensive characterization of the risk factors, clinical time course, and specific symptoms of cefepime neurotoxicity. Our objectives were to describe the spectrum of most commonly reported symptoms, risk factors for these adverse events, time frame for onset and resolution of symptoms, patient outcomes, and interventions associated with efforts to treat cefepime neurotoxicity to improve recognition and appropriate management.

## Methods

A librarian-assisted search of CINAHL and MEDLINE databases identified all English-language publications describing cefepime-associated neurotoxicity in humans from January 1980 through February 2016. Cefepime became commercially available in 1994 and this time frame was used to capture all reported cases before and after its introduction to clinical practice. Search terms included cefepime, neurotoxicity, encephalopathy, seizures, delirium, coma, non-convulsive status epilepticus (NCSE), myoclonus, confusion, aphasia, agitation, and death. Corresponding authors of included studies were contacted for any missing information, and if no response was received within several weeks, a second inquiry was performed.

Two authors (LEP and GLF) independently assessed articles for study inclusion using the Preferred Reporting Items for Systematic review and Meta-Analysis Protocols (PRISMA-P) for data reporting. All identified publications containing our criteria for participants (hospitalized patients ≥15 years of age), interventions (cefepime administration), comparisons (none required), outcomes (symptoms related to neurotoxicity, see listed search terms), and study design (all) – the PICOS criteria − were included in this systematic review [9].

Patient-specific data including demographics, creatinine, creatinine clearance, existing critical illness, central nervous system comorbidities, descriptions of cefepime neurotoxic symptoms (and timing of their onset and offset), serum and cerebral spinal fluid cefepime concentrations and their temporal relationship with cefepime administration, diagnostic tests including electroencephalography (EEG), interventions, and clinical outcomes were extracted using a prespecified data collection tool. Dosing regimens were categorized as appropriate or not relative to renal function based on estimated creatinine clearance using FDA-approved prescribing information [3]. Variables were identified as “unable to assess” if necessary patient data were unavailable and could not be obtained from the corresponding author. Co-administered medications were considered potentially neurotoxic if the FDA-approved prescribing information listed any neurotoxicity as a potential adverse reaction. Patients with reported or estimated creatinine clearance of 60 ml/min or greater were classified as having normal renal function. Those with reported chronic kidney disease (CKD), acute kidney injury (AKI), or renal insufficiency were identified as having renal dysfunction. In lieu of consensus opinion, we defined excessive serum trough cefepime concentrations as > 20 mg/L based on recent data suggesting a fivefold increase in neurologic risk when this threshold was exceeded [10]. This concentration is at least twice as high as what many authors consider as the target or therapeutic range for trough cefepime concentrations (between 5 and 10 mg/L) based on pharmacodynamic data [2, 10–12].

## Results

**Fig. 1:**
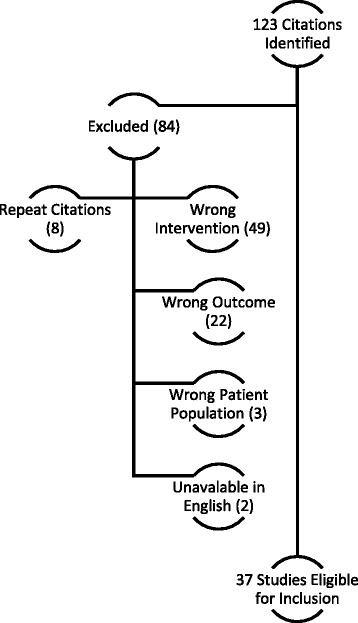
Reasons for exclusion

There were 123 citations identified; 37 were included, representing 135 patients (Fig. 1). With the exception of a prospective cohort trial [13], all studies were retrospective, including 19 single-case reports [1, 14–31], 9 case series [2, 11, 32–38], and 8 retrospective cohort studies [4, 5, 39–44]. As shown in Table 1, patients were predominantly elderly (median 69 years), had renal dysfunction (80%), and required intensive care (81%). Many assessable patients (48%) received cefepime regimens excessive for their reported renal function, but 26% were dosed appropriately, and another 26% could not be assessed due to incomplete information (Table 2). Among the 26% of neurotoxic patients thought to be dosed appropriately for renal function, 7 patients had measured serum concentrations, and all were elevated (≥20 mg/L) [2, 4]. In comparison, reported serum concentrations for patients with excessive doses ranged from 15 to 284 mg/L (median 39).

**Table 1:** Patient characteristics (n = 135)

Characteristic	Value
Median age (IQR)	69 years (54–75)
Sex (female), *n* (%)	69 (51%)
Pre-existing CNS disease, *n* (%)	11 (8%)
Cerebral vascular disease	6 (4%)
Encephalopathy	2 (1%)
Other^a^	3 (2%)
Renal function dysfunction	108 (80%)
Creatinine clearance, median (IQR)	26.5 (17–56) ml/min
History of alcohol use disorder, *n* (%)	3 (2%)
Co-administered neurotoxic drug(s), *n* (%)^b^	21 (16%)
Patient location, *n* (%)	
ICU	60 (44%)
Non-ICU	14 (10%)
Unreported	61 (45%)

*CNS* central nervous system

^a^Other pre-existing CNS diseases included encephalitis, spina bifida, dementia

^b^Reported neurotoxic medications include amikacin, ciprofloxacin, metronidazole, cytarabine, cyclosporine, tacrolimus, phenytoin

**Table 2:** Cefepime characteristics

Characteristic	Value
Cefepime dosing	
Median dose over 24 hours (IQR), g	3.5 (2–5.9)
Median frequency of dosing (IQR), hours	12 (12–24)
Appropriately dosed for renal function, *n* (%)	
No	65 (48%)
Yes	35 (26%)
Unable to assess	35 (26%)
Indication, *n* (%)	
Febrile neutropenia	11 (8%)
Pneumonia	37 (27%)
Other^a^	31 (23%)
Not reported	56 (41%)
Drug concentrations, mg/L	
Median serum, n = 21 (range)	45 (15–284)
Median trough, n = 13 (range)	38 (15–224)
Median CSF, n = 4 (range)	13 (6–18)
Median for appropriately dosed patients, n = 7 (range)	60 (22–74)
Median for inappropriately dosed patients, n = 10 (range)	39 (15–284)
Trough for appropriately dosed patients, n = 6 (range)	54 L (37–65)
Median onset of neurotoxic effects (IQR), days	4 (2–6)

*CNS* central nervous system

^a^No patients were treated for meningitis/CNS infections

Cefepime concentrations were evaluated in 21 patients (16% of cohort), with a median of 45 mg/L (range 15–284 mg/L) [1, 2, 4, 11, 16, 22, 31]. Cefepime trough concentrations were reported in 13 patients with a median concentration of 38 mg/L (range 15–224) [2, 4, 11, 22]; 12 of these 13 patients (92%) had trough cefepime concentrations greater than 20 mg/L. Cerebrospinal fluid (CSF) concentrations were reported in 4 patients (3%), with median concentration elevated at 13 mg/L (range 6–18 mg/L), resulting in a median CSF/serum ratio of 21% (range 6–45%) [2, 16, 31]. The typical CSF/serum ratio for cefepime of approximately 10% was exceeded in three of these patients [2, 31].

Neurotoxicity was identified a median of 4 days (IQR 2–6) after cefepime initiation. All patients had altered mental status. Reduced consciousness was reported in 47% of cases. Other commonly reported clinical findings included myoclonus (n = 57, 42%), confusion (n = 57, 42%), aphasia (n = 20, 15%), seizures (n = 17, 13%), and agitation (15, 11%). All patients with a documented EEG (98 patients or 73% of the total cohort) demonstrated abnormalities, with 25% experiencing NCSE, 7% myoclonic status epilepticus, 40% triphasic waves, and 39% with focal sharp waves.

Interventions for cefepime neurotoxicity included cefepime discontinuation (n = 109, 81%), reduction in dose (n = 6, 4%), treatment with one or more antiepileptic drugs (AED) (n = 48, 36%) including benzodiazepine in 44 patients (33%), or hemodialysis in 11 (8%) cases.

Clinical improvement was observed a median of 2 days (IQR 1–3) after the intervention, (Table 3). Partial or complete resolution of symptoms occurred in 39% and 50% of patients, respectively. No clinical improvement was noted in 11 patients, including 4 patients who received antiepileptic drugs (Table 3).

**Table 3:** Patient outcomes

Outcome	Value
Discharge outcome, *n* (%)	
Survived	117 (87%)
Died	18 (13%)
Received dialysis, *n* (%)	11 (8%)
Antiepileptic drug administered, *n* (%)	48 (36%)
Symptom resolution, *n* (%)	
Complete resolution of symptoms	68 (50%)
Symptom improvement	53 (39%)
No improvement	11 (8%)
Unreported/indeterminate	3 (2%)
Median time to clinical improvement, days	
All patients, n = 67 (IQR)	2 (1–3)
Emergent dialysis employed, n = 3^a^ (range)	1 (1–3)
Antiepileptic drug used, n = 26 (IQR)	2 (1–3)

^a^Only 3 of 11 patients who received emergent dialysis had reported times to improvement

## Discussion

**Fig. 2:**
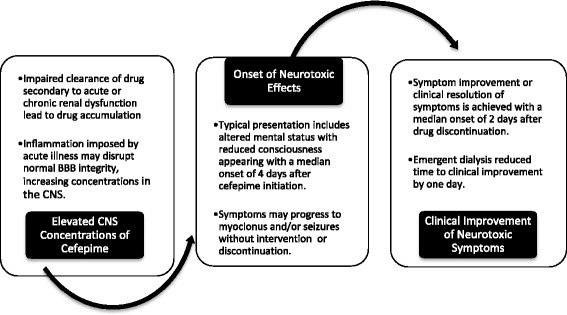
Timeline of clinical course. BBB, blood–brain barrier; CNS, central nervous system

This systematic review of cefepime neurotoxicity is the first to describe the range of symptoms (altered mental status, reduced consciousness, confusion, aphasia, myoclonus, seizures, and coma), risk factors for occurrence, and timing of onset and resolution (Table 4 and Fig. 2). Evaluated data are almost all from retrospective case reports or series from single centers, without standard data formats. The symptoms were often delayed after starting cefepime (a median of 4 days) and are common events among ICU patients, so would be easy to overlook. EEG abnormalities occurred in all monitored patients, but some of these findings are nonspecific and may reflect other causes. These events were progressive unless strategies to facilitate drug removal (e.g., drug discontinuation, drug interruption, or dialysis) or treatment interventions (antiepileptic medication administration) were initiated. A high index of suspicion is required for clinicians to ensure detection, especially in patients with predisposing risk. This review presents important information that may facilitate recognition and appropriate treatment for patients at risk.

**Table 4:** Cefepime-induced neurotoxicity – a clinical picture

Risk factors	Signs and symptoms	EEG characteristics	Treatments
- Renal dysfunction - Critical illness - Altered BBB - Older age - Drug overdose	- Altered mental status - Reduced consciousness - Confusion - Myoclonus - Aphasia - Agitation - Seizures	- Abnormalities - Tri-phasic waves - Multi-focal sharp waves - Non-convulsive SE - Generalized slowing - Myoclonic SE	- Cefepime discontinuation - Cefepime-free interval w/dose reduction - Hemodialysis - Benzodiazepine^a^

*EEG* electroencephalography, *BBB* blood–brain barrier, *SE* status epilepticus

^a^For EEG abnormalities/seizure activity associated with toxicity

Awareness of the risk factors leading to the development of cefepime-induced neurotoxicity as outlined in Table 4 is integral to the early identification of this adverse event. Our review suggests that renal dysfunction and excessive cefepime doses are major risk factors predisposing patients to cefepime neurotoxicity, though one quarter of reported patients appeared to receive appropriate dosing. Since approximately 85% of cefepime is excreted unchanged by the kidneys, renal dysfunction can dramatically increase the half-life of this antibiotic from 2 to 13 hours, prompting the need for dose adjustments [3]. Excessive cefepime exposure, defined by trough determinations greater than 20 mg/L, was reported in 12 of the 13 patients. It is important to note that even with dosage adjustments, high median and excessive trough serum concentrations were seen in this symptomatic cohort. Difficulties in accurately estimating glomerular filtration in the critically ill, leading to overestimation of renal function, wide variance in pharmacokinetics, and excessive cefepime exposure help explain these findings [7, 45].

While excessive exposure is associated with neurotoxic symptoms, characterization of this drug effect solely as a consequence of elevated drug concentrations or the suggestion that there is a threshold concentration for toxicity likely represents an oversimplification. Efforts to confirm early reports of the predictive capability of cefepime neurotoxicity when serum levels exceed 22 mg/L [4] suggest significant imprecision in this measurement with the number needed for harm ranging from 2.1 to 18.5 [46]. Other prospectively derived data suggest that neurotoxicity is associated with cefepime trough concentrations exceeding 35 mg/L [10]. These data are consistent with those found within our systematic review where 76% of patients had serum concentration determinations greater than this threshold. It appears premature to suggest that a toxic threshold concentration for cefepime has been established and that other factors such as alterations in the integrity of the BBB likely contribute to its neurotoxic potential.

Inflammatory conditions, organic acid accumulation, and renal dysfunction may predispose patients to disruptions in their BBB allowing increased CNS penetration of cefepime [47, 48]. Renal dysfunction is especially significant since it also leads to increased cefepime serum concentrations, and is associated with proteinuria, hypoalbuminemia and altered protein binding, increasing the unbound and biologically active fraction of cefepime available for entry into the CNS [8]. Three of the four patients with reported CSF concentrations had much greater CNS penetration than normal, exceeding the expected CSF/plasma cefepime concentration ratio of 10% (range = 16–45%) [31]. Median trough concentrations in appropriately dosed patients were higher than in patients receiving excessive doses; however, meaningful conclusions from these data are limited due to the small sample sizes used for comparison. Further research is needed to define the role of brain injury in the development of cefepime-induced neurotoxicity, but 11 patients included in our review had preexisting CNS diseases and 2 patients had preexisting seizure disorders [16, 20, 21, 42].

In addition to the presence of risk factors, a better understanding of the clinical course may facilitate earlier identification of cefepime-induced neurotoxicity (Table 4). It is important to stress that symptoms are often delayed, with a median onset of 4 days (IQR 2–6) after starting the drug, and trend towards a progressive course. Changes in mental status typically appear initially, but with continued cefepime administration, myoclonus and seizures can develop. EEG evaluations are almost always abnormal, but these findings are often seen with other types of encephalopathy.

The most common intervention was discontinuation (81%) or interruption of therapy with reduction of cefepime dosing (4%), sometimes in conjunction with antiepileptic medications and led to clinical resolution or improvement of symptoms in most cases. Time to symptom improvement occurred at a median of 2 days, though emergent hemodialysis, may hasten the recovery time.

Using Hill’s criteria, there is a case for a causal relationship between cefepime and neurotoxic sequelae [49]. This adverse reaction has been reported in more than 135 patients, has consistent presenting features, and is frequent in patients at risk. Some risk factors, including excessive dosing in the setting of renal disease and high serum and CSF levels, imply biologic gradient effects.

In addition, a consistent spectrum of neurotoxicity, its progressive nature with continued therapy, an appropriate and consistent temporal relationship between initiation of therapy and symptom onset, along with evidence for reversibility in a variety of different patient populations, all support a causal relationship per the Hill criteria. Importantly, there are no reports of spontaneous remission of neurotoxicity without a mitigating intervention. Furthermore, emergent dialysis, an intervention not known to directly affect nonpharmacologically mediated seizure activity, effectively removes cefepime and may result in prompt termination of epileptiform activity [1, 19, 35].

In support of biological plausibility, it is suggested that cefepime competitively binds to GABA class A receptors, impeding neurotransmission of endogenous GABA and leading to central excitation which can be treated with GABA agonists such as the benzodiazepines (a reported intervention in 44 patients in this review) [39]. This pharmacologic feature is especially important in patients with kidney disease since usual doses may result in excessive drug exposure and accumulation in the CNS.

The epileptogenic potential of beta-lactams is widely appreciated, which strengthens the likelihood of causality due to the presence of parallel evidence. A recent review of antibiotic-induced encephalopathy described three phenotypes that are distinguished by onset, epileptiform activity, psychosis, and risk factors. Cephalosporin-associated encephalopathy was consistent in its features of onset within days of antibiotic initiation, epileptiform activity with abnormal EEG findings, and being most commonly noted in patients with renal dysfunction. It is important to note that data directly comparing the neurotoxic potential of similar antibiotics are quite limited, but it appears that cefepime may impose up to a tenfold greater risk than meropenem [42, 50, 51].

### Limitations

Several limiting features of this review deserve comment. With the exception of one prospective cohort study, published data available are limited to case series, single-case reports, and retrospective studies. These study characteristics limit our systematic review to descriptive reporting and do not allow an examination of confounders related to critical illness. The observational design of reports describing interventions for cefepime neurotoxicity precludes any statement about the efficacy of any specific strategy other than to point out that symptoms did not spontaneously resolve.

The renal function of patients within our report was inconsistently defined, limiting the precision of using this metric as a risk factor for cefepime neurotoxicity. Another confounder includes the difficulties in accurately quantifying renal dysfunction using creatinine-based equations to calculate CrCl [6, 7]. This may help to explain why many neurotoxic patients had excessive serum levels despite receiving appropriate dosing regimens. Furthermore, there is no accepted definition of a “therapeutic” or “acceptable” cefepime trough concentration – though pharmacodynamic principles based on susceptibility data support a range between 5 and 10 mg/L – and published data linking a relationship between adverse events and high serum concentrations are subject to reporting bias [52]. Last, there are no data that guide an evaluation of CSF cefepime concentrations.

Distinguishing cefepime-induced neurotoxicity from many conditions present in critically ill patients remains clinically challenging, and concurrent diagnoses may confound its identification. Septic patients commonly exhibit EEG abnormalities such as triphasic waves and electrographic seizures and may exhibit encephalopathy mediated in part by the inflammatory response [53]. Adding to this complexity, sepsis itself may be a risk factor for cefepime neurotoxicity since it may alter BBB integrity and facilitate antibiotic entry into the CNS [51, 54].

Many questions remain regarding the true incidence and scope of cefepime-associated neurotoxicity. Further research evaluating cefepime concentrations in the serum and CSF may provide vital information to determine key trends [50]. Consistency in timing serum cefepime concentrations relative to dose administration and the influence of renal replacement therapy on patient outcomes will help quantify exposure risk. Prospective evaluations with more standard and rigorous datasets are needed. Observational comparative trials evaluating the incidence of neurologic symptoms and EEG monitoring in patients treated with other broad spectrum antibiotics such as piperacillin/tazobactam may provide information crucial to our understanding and safe utilization of cefepime [8, 51].

### Recommendations

Cefepime may be a modifiable risk factor for the development of acute neurologic dysfunction especially in patients with renal impairment. The use of alternative antibiotics should be considered for patients at risk, recognizing the potential for emergence of antimicrobial resistance with injudicious antibiotic choices. If substituting antibiotics is clinically inappropriate, consider EEG and clinical monitoring for changes in neurological status.

To minimize unnecessary exposure, prescribers may choose to use lower doses of cefepime in high-risk patients, but this may prove unsafe. The treatment of multi-drug resistant bacteria (MDR) often requires susceptible-dose-dependent (SDD) regimens (2 g every 8 hours with intact kidney function) to achieve adequate serum concentrations for bactericidal activity. Frequent neurologic examinations are warranted in these patients along with the recognition that these symptoms may be blunted or confounded by many co-administered medications and comorbidities. It is important for prescribers to be mindful of the limitations of estimating renal function in the critically ill since overestimations of CrCl may place patients at risk of drug accumulation and toxicity. Although cefepime serum assays are not widely available, they may be helpful to guide dosing [45].

## Conclusion

Cefepime-induced neurotoxicity occurs most commonly when inappropriately large doses are administered to patients with renal dysfunction, but up to 25% of cases occur in patients receiving proper doses, emphasizing the need to consider drug toxicity in all patients experiencing neurological deterioration. Symptoms are often delayed and progressive, but clinical improvement usually follows drug cessation or interruption, treatment of epileptiform activity, and extracorporeal drug removal. Further exploration of this adverse drug event may help us better understand which patients are at risk and how can we can safely manage this important adverse drug event.

## Abbreviations

AED: Antiepileptic drug
AKI: Acute kidney injury
BBB: Blood–brain barrier
CKD: Chronic kidney disease
CNS: Central nervous system
CrCl: Creatinine clearance
CSF: Cerebrospinal fluid
EEG: Electroencephalography
FDA: Food and Drug Administration
GABA: ϒ-aminobutyric acid
IQR: Interquartile range
NCSE: Non-convulsive status epilepticus
PRISMA-P: Preferred Reporting Items for Systematic review and Meta-Analysis Protocols
SDD: Susceptible-dose-dependent
